# Anthrax Bioterrorism: Lessons Learned and Future Directions

**DOI:** 10.3201/eid0810.020466

**Published:** 2002-10

**Authors:** James M. Hughes, Julie L. Gerberding

**Affiliations:** * Centers for Disease Control and Prevention, Atlanta, GA, USA

**Figure 1:**
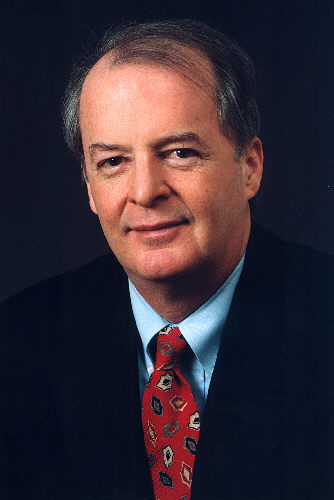
Dr. Hughes is Director, National Center for Infectious Diseases, Centers for Disease Control and Prevention, Atlanta, GA, USA.

**Figure 2:**
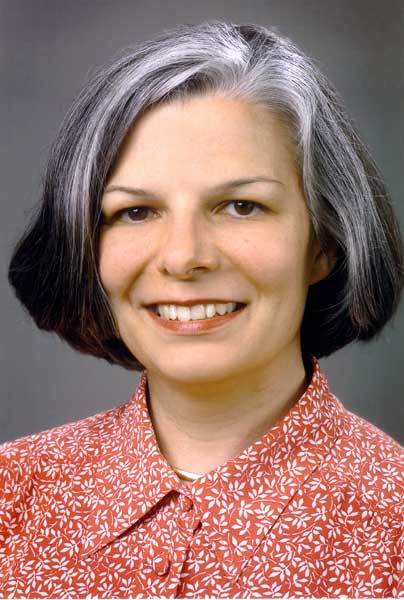
Dr. Gerbering is Director, Centers for Disease Control and Prevention, Atlanta, GA, USA.

On September 11, 2001, the United States experienced the worst terrorist attack in its history. As the nation sought to deal with this tragedy, it would face a second wave of terrorism—this time, in the form of a biological attack. The suspicion of anthrax in a patient by an astute infectious disease clinician along with capable clinical and public health laboratory staff in Florida would lead to the discovery that *Bacillus anthracis* spores had been intentionally distributed through the postal system, causing 22 cases of anthrax, including 5 deaths, and forever changing the realm of public health.

In this issue of Emerging Infectious Diseases, numerous individuals involved in the public health aspect of the anthrax investigation document their experiences. Articles describe the epidemiologic and laboratory investigations, applied research findings, environmental assessment and remediation experiences, workplace safety issues, prophylaxis and clinical care information, international aspects, and collaborations between law enforcement and public health officials. The articles also highlight the widespread efforts made to identify the source of exposure and prevent illness among those exposed. While many of the individuals involved in this effort are acknowledged in these articles, many others are not, including the large numbers of medical, public health, law enforcement, and emergency response personnel throughout the country and the world who dealt with the numerous hoaxes perpetrated in the weeks following the attack. We recognize and thank them for their heroic efforts.

This issue also provides an opportunity to review the valuable lessons we have learned from these experiences. Foremost among them is the knowledge that we cannot afford to be complacent. Throughout the Department of Health and Human Services (DHHS) as well as across other federal, state, and local agencies, we remain alert for the first evidence of a disease outbreak. Multiple systems are now in place, both in the United States and internationally, to detect initial cases. On the local level, clinicians and laboratorians play a key role in this process. Activities such as monitoring emergency room visits, pharmacy requests, calls to emergency response and poison control centers, and animal disease registries for unusual occurrences are also expanding. 

These lessons have also led us at the Centers for Disease Control and Prevention (CDC) to change the way we operate. Changes have been made within our programs, among our staff and partners, and in our coordination with other federal agencies. Many of these changes have been based on valuable input provided by public and private sector experts during numerous consultations. Terrorism response capacity is being integrated into existing infrastructures, further strengthening the foundation of public health.

The anthrax cases highlighted the importance of the “golden triangle” of response between clinicians and clinical microbiologists, the health-care delivery system, and public health officials. Steps have been taken to strengthen these and other critical linkages, including those between professionals in the human, veterinary, and public health communities and between the public health, law enforcement, and emergency response systems. 

DHHS has made available through CDC more than $918 million for state and local health departments to enhance their terrorism preparedness programs. These funds are intended to strengthen capacity to respond to bioterrorism, other infectious disease emergencies, and other urgent public health threats. Existing programs that proved invaluable during the events of last fall, such as the Laboratory Response Network for Bioterrorism (LRN) and the National Pharmaceutical Stockpile (NPS), both described in this issue in the article by Perkins et al., have also been strengthened. During the anthrax attacks, laboratories within the LRN tested more than 125,000 clinical specimens and approximately 1 million environmental specimens. The number of these specialty laboratories participating in this network has now increased to more than 100, with at least one in each state, enabling widespread testing for microbes that might be used in a terrorist attack to cause illnesses such as anthrax, tularemia, plague, and botulism. New facilities have been opened, and improvements in others are in progress or planned for the near future. The NPS has also been expanded to include additional medical supplies and personnel. State and local agencies are implementing measures to ensure the successful transport and delivery of these critical components of effective response. 

CDC has established rapid response teams composed of individuals with expertise in field operations, epidemiology, microbiology, data management, and communications. These individuals have received training to enable immediate deployment to affected areas to assist state and local efforts. The Epidemic Intelligence Service (EIS), CDC’s long-standing disease investigation training program for epidemiologists, is also undergoing changes. In addition to traditional training for rapid response to disease outbreaks, this year’s class of officers, the largest in the program’s 51-year history, is receiving specialized field training to respond to terrorist attacks that might involve the intentional release of toxic chemicals or the spread of infectious agents.

While the terrorist attacks experienced by the United States have enabled us to better prepare for, recognize, and respond to future attacks, more work needs to be done. The anthrax attack was relatively small and did not involve the use of multiple agents, multiple modes of transmission, a drug-resistant organism, transmission to animals, or global spread. The surge capacity of the health-care delivery system was not challenged. In addition, unlike some of the other threat agents, the causative organism was easily isolated in clinical laboratories; there was no risk of person-to-person transmission and no risk of vector-borne transmission. 

Planning and practice are essential to ensure an effective response to urgent public health threats. CDC has activated its emergency operations center in response to the recent outbreak of West Nile virus. During 2002, through mid-September, West Nile virus has been identified in more than 40 states and the District of Columbia and has caused more than 1,700 human cases, including more than 80 deaths. Although West Nile virus is a naturally occurring disease, because of its recent arrival in the United States many physicians are unfamiliar with the signs and symptoms suggestive of infection. As part of this response, we have provided professional education to health-care workers, evaluated the quality of laboratory processing of suspected samples, and streamlined communication—all critical components for responding to this outbreak and for identifying ways to improve our capabilities for addressing future emergencies.

Integral to planning is education. Health-care workers, particularly physicians and nurses, need training about the clinical aspects of diseases that may result from the use of biological agents. As has been evident in many recent investigations (e.g., hantavirus pulmonary syndrome, West Nile virus meningitis/encephalitis, anthrax), alert and knowledgeable clinicians and laboratorians are vital to disease surveillance efforts and recognition of new diseases and syndromes. Education of the public regarding the signs and symptoms of diseases associated with infectious agents is also essential. CDC will continue to work with partners in clinical medicine and public health to provide training for health-care providers and microbiologists and to seek innovative ways to disseminate information to the public.

The efforts of this past year to improve terrorism response capacities have been widespread, crossing multiple levels and types of organizations and professions as well as international borders. Within the public health system, we intend to continue these efforts, strengthening existing and establishing new partnerships with diverse agencies, specialties, and disciplines. While we believe that these efforts will enable us to respond aggressively and effectively in the event of a future bioterrorist attack, we acknowledge that inherent to terrorism is the unknown. As was evident in the anthrax investigation, we must learn as we go, adapting our responses as new information becomes available and continuing to strive for excellence in our science, service, systems, and strategies. Investments made in the public health system to increase preparedness to address the threat of bioterrorism will also pay dividends in preparedness to confront the next influenza pandemic, other emerging infectious diseases, and other threats to public health.

